# OpenDock: a pytorch-based open-source framework for protein–ligand docking and modelling

**DOI:** 10.1093/bioinformatics/btae628

**Published:** 2024-10-21

**Authors:** Qiuyue Hu, Zechen Wang, Jintao Meng, Weifeng Li, Jingjing Guo, Yuguang Mu, Sheng Wang, Liangzhen Zheng, Yanjie Wei

**Affiliations:** Shenzhen Institute of Advanced Technology, Chinese Academy of Sciences, Shenzhen, 518000, China; University of Chinese Academy of Sciences, Beijing, 100049, China; School of Physics, Shangdong University, Jinan, 250100, China; Shenzhen Institute of Advanced Technology, Chinese Academy of Sciences, Shenzhen, 518000, China; School of Physics, Shangdong University, Jinan, 250100, China; Centre in Artificial Intelligence Driven Drug Discovery, Faculty of Applied Sciences, Macao Polytechnic University, Macao SAR, 999078, China; School of Biological Sciences, Nanyang Technological University, Singapore 637551, Singapore; Shanghai Zelixir Biotech Co. Ltd, Shanghai, 201203, China; Shenzhen Zelixir Biotech Co. Ltd, Shenzhen, 518107, China; Shenzhen Institute of Advanced Technology, Chinese Academy of Sciences, Shenzhen, 518000, China

## Abstract

**Motivation:**

Molecular docking is an invaluable computational tool with broad applications in computer-aided drug design and enzyme engineering. However, current molecular docking tools are typically implemented in languages such as C++ for calculation speed, which lack flexibility and user-friendliness for further development. Moreover, validating the effectiveness of external scoring functions for molecular docking and screening within these frameworks is challenging, and implementing more efficient sampling strategies is not straightforward.

**Results:**

To address these limitations, we have developed an open-source molecular docking framework, OpenDock, based on Python and PyTorch. This framework supports the integration of multiple scoring functions; some can be utilized during molecular docking and pose optimization, while others can be used for post-processing scoring. In terms of sampling, the current version of this framework supports simulated annealing and Monte Carlo optimization. Additionally, it can be extended to include methods such as genetic algorithms and particle swarm optimization for sampling docking poses and protein side chain orientations. Distance constraints are also implemented to enable covalent docking, restricted docking or distance map constraints guided pose sampling. Overall, this framework serves as a valuable tool in drug design and enzyme engineering, offering significant flexibility for most protein–ligand modelling tasks.

**Availability and implementation:**

OpenDock is publicly available at: https://github.com/guyuehuo/opendock.

## 1 Introduction

Molecular docking algorithms and their applications for ligands are valuable tools for understanding drug–target interactions, discovering potential drug molecules, designing new compounds, repurposing existing drugs, and designing highly active enzymes (Anderson 2003, Grinter and Zou 2014, Macalino *et al.* 2015, Luo *et al.* 2024). The broad range of applications of molecular docking necessitates the continuous development of accurate protein–ligand modelling tools and scoring functions (Agamah *et al.* 2020, Paul *et al.* 2021, Meli *et al.* 2022).

Recent research has primarily focused on the accuracy of scoring functions, evaluated using various metrics such as scoring power, ranking power, docking power, and screening power (Su *et al.* 2018). Early scoring methods, which were empirical, statistical, or molecular mechanics-based, often exhibited unsatisfactory docking and screening performance (Wójcikowski *et al.* 2017, Li *et al.* 2019, Pinzi and Rastelli 2019, Meli *et al.* 2022). In the early 2010s, machine learning-based scoring functions such as RF-score (Ballester and Mitchell 2010) and its derivatives (Wójcikowski *et al.* 2017), as well as NNscore (Durrant and McCammon 2011), were developed by fitting experimentally determined protein–ligand binding affinities using crystal structures of the complexes. Later, deep learning algorithms—including convolutional neural network-based models (Stepniewska-Dziubinska *et al.* 2018, Zheng *et al.* 2019b, Wang *et al.* 2021, Ye *et al.* 2024), topological data analysis-based models (Liu *et al.* 2021), and graph-based models (Nguyen and Wei 2019, Jiang *et al.* 2022)—were introduced to predict binding affinity and pose quality, as measured by root mean squared deviations (RMSD) between the docking pose and the experimental native pose (Bao *et al.* 2021, McNutt *et al.* 2021, Dhakal *et al.* 2022, Zheng *et al.* 2022, Wang *et al.* 2023, Zhu *et al.* 2023). Some of these models require numerous external packages for feature extraction or model inference, complicating their use and precluding their application in ligand pose optimization when gradient computations are not feasible. Most of these scoring functions, however, have rarely been explicitly combined with sampling strategies for protein–ligand docking and modelling. In contrast, DeepRMSD (Wang *et al.* 2023) computes features directly from protein–ligand atom coordinates and can implement minimization-based optimization to refine ligand coordinates in Cartesian space, making it a useful scoring function for molecular docking and modelling.

Traditional protein–ligand docking applications use various sampling strategies to generate numerous ligand conformations (or docking poses) and protein side-chain orientations when protein flexibility is considered (Dias *et al.* 2008, Pagadala *et al.* 2017, Pinzi and Rastelli 2019, Zhang *et al.* 2022, Wang *et al.* 2024). For example, AutoDock Vina is one of the most popular protein–ligand docking tools. AutoDock Vina (Trott and Olson 2010) utilizes an empirical scoring function comprising five intermolecular terms and a torsional normalization term for ligand entropy estimation. For sampling, it implements a grid-based search algorithm that performs a Monte Carlo-based search with LBFGS-based (Moritz *et al.* 2016) local optimization along the grid-based energy field. Another docking tool, Glide (Friesner *et al.* 2004), a commercial software, incorporates a hybrid scoring function combining a simple molecular mechanics energy function with empirical scoring terms. The scoring function evaluates factors such as Van der Waals interactions, Coulombic electrostatic interactions, hydrogen bonding, and hydrophobic interactions. Glide usues a combination of sampling strategies, including systematic and stochastic searches. It uses a hierarchical docking approach that begins with fast ligand conformational sampling using a systematic search, followed by a more refined sampling phase using a stochastic search algorithm. Recent deep learning-based docking applications, such as Gnina (McNutt *et al.* 2021), adopts the AutoDock Vina sampling strategy with deep learning-based scoring functions for both docking pose RMSD estimation and binding affinity prediction. Other methods, such as EquiBind (Stärk *et al.* 2022) and TankBind (Lu *et al.* 2022), leverage neural networks to extract complex features and capture essential global and overall binding interactions in blind docking scenarios (Zhang *et al.* 2024). More recent works, such as AlphaFold3 (Abramson *et al.* 2024) and RoseTTAFold (Krishna *et al.* 2024) focus on the protein–ligand full-atom structure prediction, demonstrating the significant accuracy improvement comparing to traditional docking method AutoDock Vina.

It is now well recognized that scoring and sampling are the two most important components for molecular docking (Dias *et al.* 2008, Pagadala *et al.* 2017). The development of balanced scoring functions for both docking and screening tasks is of vital importance (Shen *et al.* 2023). Incorporating accurate scoring functions with efficient sampling strategies would therefore significantly improve protein–ligand modelling. However, most docking tools are optimized for high speed in large-scale library screening (Li *et al.* 2012, Alhossary *et al.* 2015), making them difficult to redesign or modify. Consequently, they are not ideal candidates for developing flexible protein–ligand modelling methods.

To address these issues, we introduce OpenDock, an open-source, PyTorch-based protein–ligand docking framework designed for flexible protocol building and method development. In this framework, the sampling and scoring modules are defined in a separate, objective-oriented manner, allowing users to build their own sampling and scoring methods. Several common sampling strategies, such as the Monte Carlo method (MC) (Kroese *et al.* 2014), genetic algorithms (GA) (Mirjalili and Mirjalili 2019), and particle swarm optimization (PSO) (Wang *et al.* 2018), are readily available for conformation sampling. For improved performance in local optimization of docking poses, optimization methods such as Limited-memory Broyden-Fletcher-Goldfarb-Shanno (L-BFGS) (Zhu *et al.* 1997), Adam (Kingma and Ba 2014), and stochastic gradient descent (SGD) (Bottou 2010) are also included. Moreover, both the original AutoDock Vina scoring function and the deep learning-based scoring function DeepRMSD (Wang *et al.* 2023) are implemented for both global sampling and local optimization, and external scoring methods (Shen *et al.* 2022, Zheng *et al.* 2022) and user-defined scoring functions can also be utilized in the docking process. What’s more, various constraints can be incorporated to support restricted docking tasks, such as covalent docking and enzyme-substrate modelling. The performance of different combinations of sampling and scoring methods is evaluated based on re-docking and cross-docking benchmarks. Overall, OpenDock is a versatile protein–ligand docking framework suitable for method development and complex protein–ligand modelling tasks. The source code for OpenDock is available in the GitHub repository: https://github.com/guyuehuo/opendock, and documentation and tutorials can be found here: https://opendock-readthedocs.readthedocs.io.

## 2 Materials and methods

### 2.1 Ligand and receptor flexibility representations

The internal coordinate method can be used to reduce the degrees of freedom in a molecule, allowing for more efficient molecular simulations and conformational searches (Trott and Olson 2010, Wang *et al.* 2023). By using internal coordinates, the molecule can be divided into different frames, within each of which the relative positions and angles are kept invariant. Consequently, it is only necessary to explicitly describe the essential degrees of freedom of the molecule (or the protein side-chains) by a few degrees of frames. For instance, in AutoDock Vina (Trott and Olson 2010) and AutoDockFR (Ravindranath *et al.* 2015), ligands are described as a (6+k)-dimensional vector to facilitate translation, rotation, and adjustment of rotatable angles, thereby enabling the representation of ligand motion. In contrast, the protein maintains the rigidity of the backbone, while the side-chains are flexibly modelled through the adjustment of up to four torsional angles. This method significantly reduces the system’s degrees of freedom, enhancing modelling efficiency and shortening computational time.

Within the OpenDock framework, we adopt the same approach, utilizing the manipulation of the ligand’s conformational vector (6+k dimensions) for sampling, while optimizing and sampling amino acid side-chains through vectors of up to four angles for each amino acid. The input conformation is initially defined by such a pose vector and can be further updated. The updated vector can then be converted back into Cartesian coordinates using Rodrigues’ rotation formula (Piña 2011, Dai 2015) for scoring function-based energy or potential calculations (Wang *et al.* 2023).

### 2.2 Minimization strategies

**Figure 1.**
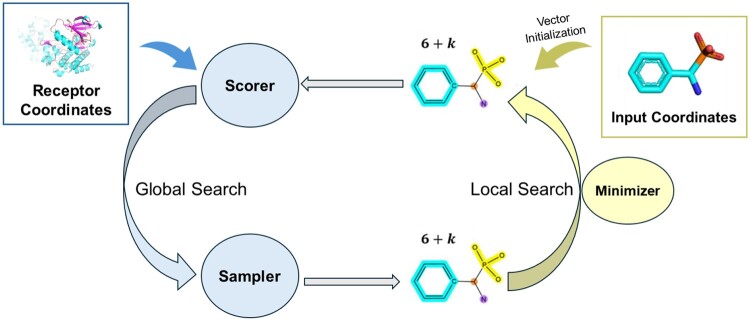
The optimization process of ligand coordinates in OpenDock. The ligand is represented as a 6+k dimensional vector. Initially, a global search based on the sampling strategy generates a new vector. This is followed by local optimization, where the score is used as the loss function for the neural network to refine the vector further.

For the local optimization strategy, we have adopted an approach utilizing neural networks, where multiple optimizers such as Adam and L-BFGS can be selected. Building on our previous work (Wang *et al.* 2023), we represent the conformation of ligand ligands using a 6+k dimensional vector. As illustrated in Fig. 1, during the optimization process of ligand conformations, the initial coordinates of heavy atoms are derived from the ligand vector. The current conformation is then scored based on the coordinates of heavy atoms using a differentiable scoring function (e.g. Vinascore (Trott and Olson 2010), DeepRMSD (Wang *et al.* 2023), with Vinascore as the default). Using the score as the loss function, the ligand vector is optimized through a minimizer (L-BFGS, SGD, or Adam). This process iterates for a specific number of steps, resulting in an updated ligand vector.

### 2.3 Sampling strategies

In protein–ligand molecular docking algorithms, sampling algorithms are used to search for and explore the potential binding modes between the receptor and the ligand (Grinter and Zou 2014, Pagadala *et al.* 2017, Pinzi and Rastelli 2019). Their primary objective is to sample the conformational space to identify the optimal binding mode.

This can be achieved through movements, rotations, or adjustments of the angles of rotatable bonds in the ligand and the side-chains of the protein on a large scale for global pose sampling. Subsequently, the sampling algorithm evaluates the energy or score of the newly generated binding complex to determine its likelihood of representing an ideal binding mode. For instance, in the MC method, if the energy or score of the new conformation is lower, indicating a more favorable binding, it is accepted as the current best binding mode. If the energy or score is higher, the conformation may be discarded or accepted with a certain probability based on the Metropolis algorithm (Bhanot 1988, Kroese *et al.* 2014). Sampling algorithms can adopt various strategies and techniques as needed. Other sampling algorithms include GA, molecular dynamics simulations (MD) (Karplus and Petsko 1990, Hollingsworth and Dror 2018, Zheng *et al.* 2019a), PSO, and others. These algorithms, along with local optimization steps, adaptively search the binding space and continuously optimize the binding mode during the simulation process.

Unlike traditional molecular docking applications, which implement only a predefined sampling strategy, OpenDock supports multiple sampling methods (Table 1).

**Table 1.** Sampling strategies implemented in OpenDock.

Name	Description	OpenDock class
Monte Carlo	Monte Carlo based sampling	MonteCarloSampler
Genetic Algorithm	The degree of freedoms are treated as chromosomes	GeneticAlgorithmSampler
Particle Swarm Optimization	Simulating cooperation and competition among individuals	ParticleSwarmOptimizer

### 2.4 Scoring functions and their implementation

The scoring function for protein–ligand docking is a mathematical function that evaluates the affinity and binding pattern (Su *et al.* 2018, Li *et al.* 2019, Meli *et al.* 2022). It is typically based on principles of physical chemistry and statistical analysis and is used to predict the interaction capabilities between proteins and ligands. The scoring function can be designed based on different methods and principles. Common scoring functions include: empirical-based scoring functions (Eldridge *et al.* 1997, Trott and Olson 2010, Guedes *et al.* 2018), statistical analysis-based scoring functions (Gohlke and Klebe 2001, Dittrich *et al.* 2018), force field based scoring functions (Yin *et al.* 2008), and ML or DL-based scoring functions (Stepniewska-Dziubinska *et al.* 2018, Liu *et al.* 2021, McNutt *et al.* 2021, Paul *et al.* 2021, Zheng *et al.* 2022, Wang *et al.* 2023).

The scoring function in molecular docking is utilized to facilitate directed conformational searching and assist in identifying ligand poses, as well as protein side-chain conformations, that closely resemble the crystal complex structure (native state) (Su *et al.* 2018, Li *et al.* 2019). Through iterative sampling and scoring, the conformational space can be explored either through exhaustive traversal or random search. In certain methods, differentiable scoring functions can guide local optimization (Trott and Olson 2010) in conjunction with global searching (Alhossary *et al.* 2015) of ligand poses, enabling more efficient and accurate conformational sampling.

Within our framework, the traditional scoring function Vinascore (Trott and Olson 2010) is supported (Table 2), along with deep learning (DL) and machine learning (ML) based scoring functions. Furthermore, users have the flexibility to customize scoring functions, such as the Vina-style methods implemented in Smina (Masters *et al.* 2020), or to develop novel scoring algorithms to enhance the effectiveness of molecular docking and virtual screening.

**Table 2.** Support scoring functions in OpenDock.

Name	Description	Compatible with samplers	Compatible with minimizers
Vinascore	Empirical SF	Yes	Yes
DeepRMSD	DL-based SF	Yes	Yes
OnionNet-SFCT	ML-based SF	Yes	Yes
RTMscore	DL-based SF	Yes	Yes
Customized SF	User defined SF	Yes	Depends
Hybrid SF	The combination of different SFs	Yes	Depends
Constraint SF	Different types of constraints	Yes	Yes

### 2.5 Distance constraints

The purpose of setting intermolecular constraints in molecular docking is to restrict the relative position and orientation between a ligand and a protein, thereby guiding and optimizing the search for binding modes based on a predefined hypothesis (Ouyang *et al.* 2013). Intermolecular constraints can leverage known structural information to guide the molecular docking process in covalent-based docking (Ouyang *et al.* 2013) and pharmacophore-based docking (Hindle *et al.* 2002). To achieve efficient constraints, harmonic or more sophisticated constraints can be defined on bond lengths, bond angles, and dihedral angles to ensure compliance with known bond lengths and angles (Wang and Dokholyan 2019). By setting appropriate intermolecular constraints, the search space can be effectively narrowed, thereby improving the efficiency and accuracy of molecular docking and aiding researchers in gaining better insights into molecular interactions.

In OpenDock, intermolecular constraints can be implemented using the *Constraint* class. This *Constraint* class extends the functionality of a scoring function, allowing for the control of molecular motion through the adjustment of constraint positions and strengths using harmonic functions and corresponding force constants, as utilized in many molecular modelling tools (Hindle *et al.* 2002, Rohl *et al.* 2004, Ouyang *et al.* 2013, Krippahl and Barahona 2015, Wang and Dokholyan 2019). Such constraints can be employed for modelling substrates and key catalytic amino acids in enzyme-substrate systems, as well as for achieving covalent docking (Ouyang *et al.* 2013).

The default constraint type in the current framework is limited to distance constraints or combinations of distances, but future versions will incorporate more types of constraints. In addition to the types of constraints, the way constraints are applied can also be implemented through various functions, such as the *upper_wall* function to limit the upper distance, the *lower_wall* function to set a lower distance limit, or the *wall* function to constrain the distance within a range. Meanwhile, users have the flexibility to define their own constraints, such as angle constraints or deviations from the initial state of ligands. This customized constraint scheme provides a highly useful and flexible modelling approach. Regarding how to add new constraint types, we have provided a detailed tutorial (available at https://opendock-readthedocs.readthedocs.io/en/latest/add_custom_costr.html) that explains the process, using angle constraints as an example. Other types of constraints, such as dihedral angle constraints and hydrogen bond constraints, can be implemented in a similar manner.

## 3 Results and discussion

### 3.1 The framework of OpenDock

AutoDock Vina (Trott and Olson 2010) is a widely used protein–ligand docking software. Its input consists of a rigid receptor (or sometimes flexible side-chains), a flexible ligand, and a docking box to restrict the conformational space to a specific binding site. This setup facilitates the calculation of a grid-based energy table (Morris *et al.* 2009) to accelerate the subsequent scoring steps. The output includes predicted conformations and binding energies. OpenDock shares similar strategies with AutoDock Vina in basic usage, but introduces new definitions in the utilization and combination of scoring functions, sampling strategies, and local optimization.

**Figure 2.**
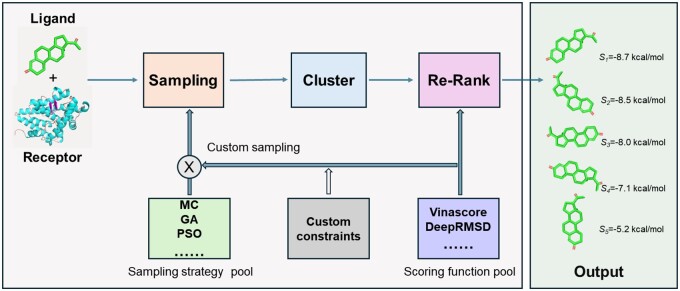
Workflow of OpenDock. After inputting ligand and receptor files, the user selects sampling strategies and scoring functions. Custom constraints can be applied during sampling. Once sampling is complete, a series of ligand poses is outputted. After clustering, the user can select scoring functions for post-ranking, and the final docked ligand is outputted.

Figure 2 illustrates the overall workflow of OpenDock. During initialization, users can customize scoring functions, sampling strategies, and define constraints through the constraint class. A hybrid scoring function (referred to as *HybridSF*) can also be compiled from multiple scoring functions. By setting different combinations of scoring functions with sampling strategies, the weights of the scoring functions are explored to achieve higher accuracy. Once users select and define the scoring functions, sampling strategy, and constraints, OpenDock explores the ligand conformational space according to the sampling strategy. After sampling, clustering removes redundant poses using a default 1.0 Å RMSD cutoff. In post-processing, users can select new scoring functions to re-rank the poses, save them to the specified output folder, and display predicted binding free energies in the output file.

**Figure 3.**
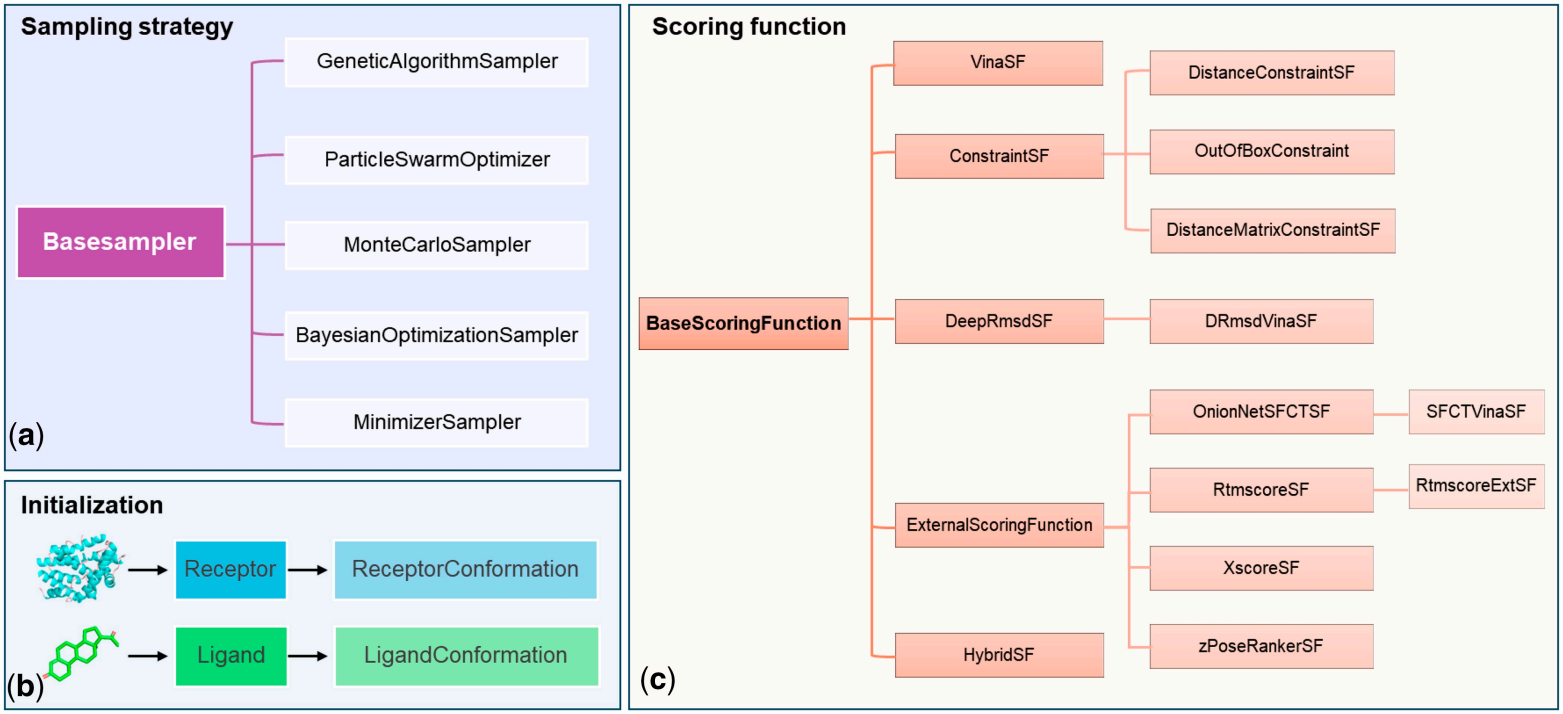
Class diagram. Revealed the implementation methods of various sampling strategies and scoring functions, along with the class relationships that implement them. The left-to-right arrangement represents class inheritance relationships.

Figure 3 illustrates the relationships between various classes in OpenDock, revealing the connections between different sampling strategies and scoring functions. Of course, there are currently various popular molecular docking tools(Rarey *et al.* 1996, Jones *et al.* 1997, Friesner *et al.* 2004, Trott and Olson 2010, Koes *et al.* 2013, McNutt *et al.* 2021, Stärk *et al.* 2022, Lu *et al.* 2022, Corso *et al.* 2024), including machine learning-based methods. The characteristics, advantages, and disadvantages of several docking tools, including OpenDock, are listed in Table 3, with detailed descriptions available in Supplementary material Part 3. Compared to other tools, OpenDock offers a high degree of flexibility and supports custom constraint docking. However, it faces significant computational overheads that affect its efficiency.

**Table 3.** Sampling strategies, scoring functions, advantages and disadvantages of multiple docking tools.

Tool	Sampling strategy	Scoring function	Advantages	Disadvantages
AutoDock Vina	Monte Carlo	Empirical	Fast, easy-to-use	Lack of flexibility
Glide	Grid-based	Empirical	High precision	Time-consuming
Smina	Monte Carlo	Empirical	Customizable	Needs tuning
FlexX	Incremental	Empirical	Good for rigid docking	Lack of flexibility
Gold	Genetic algorithm	ChemScore, GoldScore	Effective for diverse ligands	Time-consuming
**OpenDock**	Multiple sampling strategies	Multiple scoring functions	Flexibility, custom constraint	Time-consuming
Gnina	DL-enhanced MC	CNN-based	Uses 3D convolutions	GPU dependent
EquiBind	Flexible ligand sampling	DL-based	Flexible binding models	Less validated
TankBind	Distance-based ligand optimization	DL-based	Targets tough proteins	Resource intensive
DiffDock	Diffusion model	Physics-informed DL	Innovative, handles changes	High computational needs

In the upcoming tests, multiple datasets will be used to validate redocking, cross-docking, and blind-docking scenarios (see Supplementary material Part 1 for details). After an initial parameter exploration and comparison with AutoDock Vina (Supplementary material Part 2), using a learning rate of 0.1, local optimization steps of 5, and MC sampling exhaustiveness set to 8 (with a maximum of 32). We proceeded to customize combinations of different scoring functions and sampling strategies. These combinations were based on the options outlined in Tables 1 and 2 for redocking and cross-docking scenarios.

### 3.2 Multiple sampling strategies in OpenDock

After introducing and thoroughly studying the characteristics of three sampling strategies (Supplementary material Part 4), next, we explored docking performance using different sampling strategies. The sampling process and post-processing scoring function default to Vinascore.

**Figure 4.**
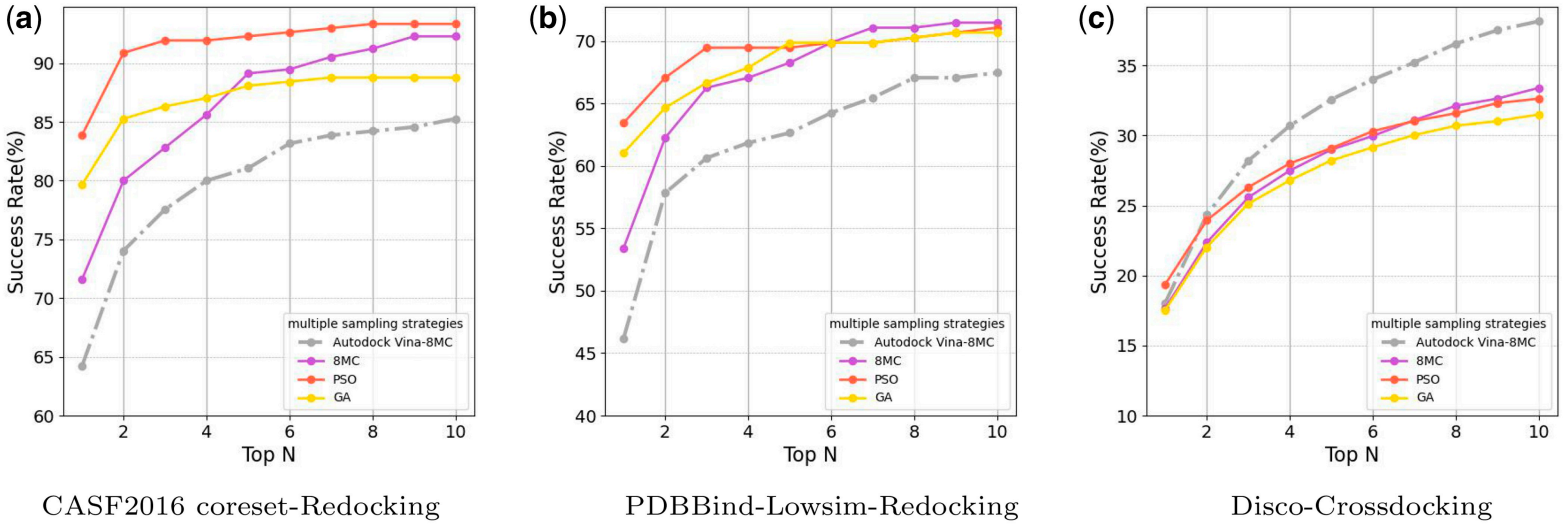
Comparison of multiple sampling strategies, with both sampling process and post-processing utilizing the Vinascore. 8MC stands for Monte Carlo sampling with an exhaustiveness setting of 8. Top N is the percentage of targets ranked above or at N with an RMSD value <2 Å. (a) CASF2016 coreset-Redocking, (b) PDBBind-Lowsim-Redocking, (c) Disco-Crossdocking.

Comparing three sampling strategies using AutoDock Vina results as a reference, with MC sampling’s exhaustiveness set to 8 (8MC), Fig. 4 shows that in redocking tasks, both GA and PSO sampling exhibit similar performance to MC sampling in the top-10 success rate, but significantly improve the success rate for the top-1 poses. Particularly for PSO sampling, in the CASF2016 core set, the top-1 success rate reaches approximately 84%, even with shorter sampling times. The improvement in top-1 performance by GA and PSO can be attributed to multiple ligands learning from each other during the sampling process, allowing them to learn better and faster within the same or even shorter time frame compared to individual MC sampling. This underscores the significant impact of effective sampling strategies on molecular docking and demonstrates the feasibility of local optimization akin to more efficient minimizers. In cross-docking tasks, AutoDock Vina slightly outperforms the three sampling strategies in the top-10 success rate, while the success rates of the three sampling strategies remain similar.

It is worth noting that there is still considerable room for exploration in sampling strategies. Undoubtedly, through further exploration, it is possible to achieve improved results in shorter periods of time.

### 3.3 Multiple scoring functions in OpenDock

OpenDock supports the use of various scoring functions and allows for custom combinations of multiple scoring functions through the *HybridSF* class. Given the time-consuming nature of using the DL-based scorer DeepRMSD (Wang *et al.* 2023), the default scoring function during the sampling process is Vinascore. After clustering is completed, post-processing can use scoring functions such as DeepRMSD or a combination of DeepRMSD and Vina to re-rank the clustered conformations. In practice, the weight of the DeepRMSD scoring within the DeepRMSD+Vina hybrid scoring function was set to 0.5 and 0.8, respectively.

**Figure 5.**
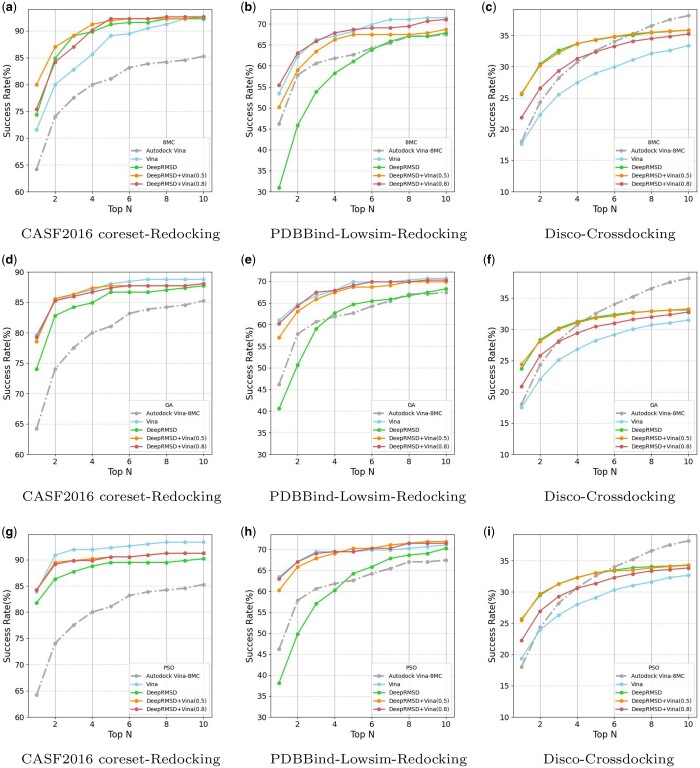
Performance of using multiple scoring functions with different sampling strategies on redocking and crossdocking tasks. (a–c) Sampling strategy for 8MC, (d–f) sampling strategy for GA, (g–i) sampling strategy for PSO. Different scoring functions were employed for post-processing. DeepRMSD+Vina(0.5) indicates that the weight of Vinascore is set to 0.5. Top N is the percentage of targets ranked above or at N with a RMSD <2 Å.

Using the results of AutoDock Vina as a reference, with its exhaustiveness set to 8, OpenDock adopts an MC sampling strategy with the default exhaustiveness value of 8 (8MC). The results using other exhaustiveness values can be found in the Supplementary material Part 8. For sampling strategies such as GA and PSO, various scoring functions are also used for post-processing. As seen in Fig. 5, post-processing with DeepRMSD and DeepRMSD+Vina scoring functions on the CASF2016 core set dataset effectively improves the top-1 docking success rate, with OpenDock’s performance significantly surpassing that of AutoDock Vina. The best results were obtained using DeepRMSD+Vina with the Vina weight set to 0.5, which is consistent with previous experimental outcomes (Wang *et al.* 2023). The use of DeepRMSD on the PDBBind-Lowsim dataset showed poor performance, which might be related to the model’s generalization ability (Su *et al.* 2020). In the cross-docking tasks on the Disco dataset, post-processing re-ranking with DeepRMSD and DeepRMSD+Vina scoring functions also effectively increased the docking success rate.

It is worth noting that in redocking tests, OpenDock significantly outperforms AutoDock Vina. However, in cross-docking validation, their performance is similar. This difference may be because, in redocking, the optimal ligand conformations are known, allowing the scoring function to quickly guide conformations to these positions. In cross-docking, the accuracy limit is constrained by the Vinascore scoring function, explaining the similar performance of both tools.

### 3.4 Blind docking on the DockGen dataset

To comprehensively and rigorously evaluate OpenDock, we will utilize the novel-pocket dataset—DockGen, which comprises 189 cases. The performance of OpenDock will be compared in terms of accuracy and computational efficiency against mainstream sampling-based docking software, such as AutoDock Vina, Smina (Koes *et al.* 2013), Gnina (McNutt *et al.* 2021), as well as prominent machine learning-based tools, including Equibind (Stärk *et al.* 2022), Tankbind (Lu *et al.* 2022), and DiffDock-L (Corso *et al.* 2024).

When using search-based docking tools to perform blind docking, it is first necessary to predict the binding pocket’s location. To this end, we utilized P2Rank (Krivák and Hoksza 2018) for pocket prediction on the proteins before proceeding with molecular docking. By using the crystal structure conformation as the ligand input, we yielded the results presented in Table 4, which include docking success rates and computational time.

**Table 4.** Top-1 RMSD performance of different methods on the DOCKGEN benchmarks with crystal structure input.

Method	% <2Å	% <5Å	CPU(s)	GPU(s)
P2rank+AutoDock Vina(8)^a^	17.5	36.0	15.1	–
P2rank+AutoDock Vina(32)^a^	17.5	36.5	26.3	–
P2rank+Smina(8)^a^	16.4	34.4	**13.7** ^b^	–
P2rank+Smina(32)^a^	18.0	37.0	29.7	–
P2rank+Gnina(8)^a^	16.9	32.3	137.3	–
P2rank+Gnina(32)^a^	**22.8** ^b^	37.0	147.0	–
P2rank+OpenDock-MC(8)^a^	19.0	37.6	197.1	–
P2rank+OpenDock-MC(32)^a^	20.6	39.7	884.2	–
P2rank+OpenDock-GA(8)^a^	18.5	35.0	368.4	–
P2rank+OpenDock-GA(32)^a^	21.7	36.5	1384.2	–
P2rank+OpenDock-PSO(8)^a^	18.0	32.8	315.8	–
P2rank+OpenDock-PSO(32)^a^	22.2	36.0	1214.7	–
Equibind	0.0	4.8	–	**0.04** ^b^
Tankbind	0.5	16.9	–	0.7
DiffDock-L	21.2	**54.5** ^b^	246.3	25

The values in parentheses refer to the level of exhaustiveness of the search; for example, (8) indicates that the exhaustiveness was set to 8. Smina and Gnina run according to default settings.

a Assign 24 CPU cores to each task.

b Bold indicates optimal performance.

From the results in Table 4, where the crystal structure conformation was used as input, traditional search-based tools such as AutoDock Vina and Smina demonstrated good accuracy and excellent computational efficiency. OpenDock’s three sampling strategies showed slightly higher success rates compared to AutoDock Vina and Smina, though at the expense of computational efficiency. Among machine learning-based tools, DiffDock-L performed well, particularly leading in the success rate for RMSD below 5 Å, though the time required for its execution on a CPU was significantly higher than the GPU-based execution reported in the literature. As for EquiBind and TankBind, their success rates were far behind those of the other tools.

For a comprehensive evaluation in the docking tests, we also assessed the docking performance using ligand conformations that deviate significantly from the crystal structure, generated by AutoDock Vina. This approach aimed to evaluate the robustness of the docking tools under conditions where the ligand input is less accurate. In this scenario, DiffDock-L, Gnina, and OpenDock exhibited a certain degree of performance decline. Detailed results can be found in Supplementary material Part 7.

### 3.5 Conformation restriction and local optimization

In OpenDock, molecular constraints can be implemented through the *Constraint* class to guide and optimize the search for binding modes by restricting the relative position and orientation between the ligand and receptor. Users can customize various constraints. Here, we will impose constraints on the distances between atoms of the ligand and receptor. Assuming the native distances are known, the goal is to optimize a ligand conformation under these constraints and other scoring functions to closely resemble its native state.

**Figure 6.**
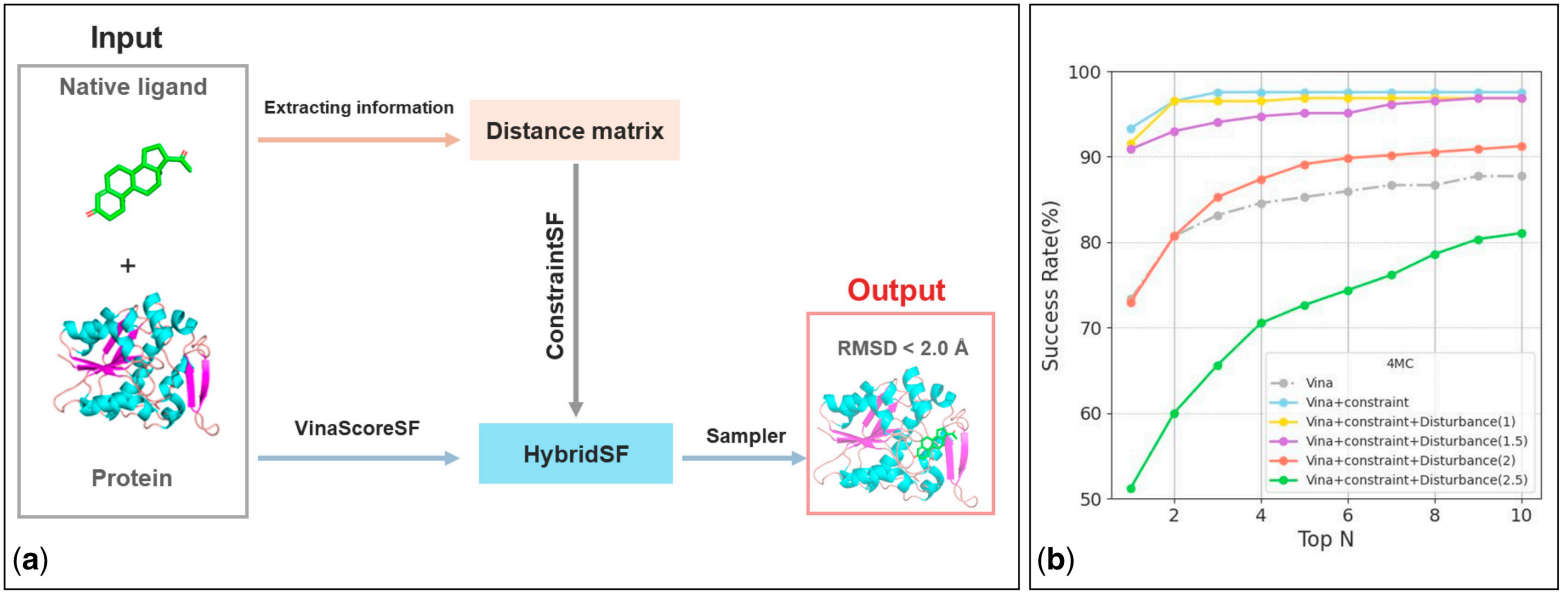
(a) Workflow for implementing constraints: experimentally obtained protein and ligand crystal structures provide valuable information, such as distance matrices, specific atom pair distances, and angle information. This information forms a constrained scoring function, which is combined with Vinascore to create an enhanced scoring function. Sampling is then conducted using this enhanced function. (b) Comparison on the CASF2016 Coreset Dataset: The 4MC method is used for redocking tests, comparing the Vina+constraint scoring function against the Vina scoring function alone. Disturbance(*x*) denotes adding Gaussian noise with a mean of *x* to the natural distance matrix.

The process of implementing constraints is illustrated in Fig. 6a. From the ligand–protein interaction, the distance matrix between atomic pairs can be extracted (from their native protein–ligand crystal complex structure) and defined as a constraint scoring function. This function is combined with Vinascore to create a new scoring function for docking.

In terms of specific implementation, we input a distance matrix of the native ligand-receptor atomic distances, referred to as the native distance matrix, which can also be externally provided if predicted (Lu *et al.* 2022, Stärk *et al.* 2022). Using the *Constraint* class, the summed squared difference between the current ligand-receptor distance matrix and the native distance matrix (from the crystal structure) is used as the score to implement the constraint scoring function. The *HybridSF* class combines this constraint scoring function with Vinascore, setting the weights of both scoring functions to 0.5.

Redocking experiments were performed on the CASF2016 core set dataset, using Monte Carlo sampling with an exhaustiveness set to 4 (four rounds of MC sampling). Both the sampling process and post-processing used the Vina+constraint scoring function. As shown in Fig. 6b, given a native distance matrix, the use of constraints significantly improves docking success rates, approaching 100%, which fully demonstrates the effectiveness of the constraints.

In practice, obtaining the native distance matrix is unlikely. Therefore, we added standard Gaussian perturbations (Hoel *et al.* 1954) with different variations to the distance matrix. The results show that with a perturbation mean of 2 Å, the outcomes are comparable to using only the Vina scoring function. This is related to our defined docking success criteria. Clearly, when the perturbation mean exceeds 2 Å, the docking results tend to deteriorate.

### 3.6 Hands on a protein–ligand docking protocol

**Figure 7.**
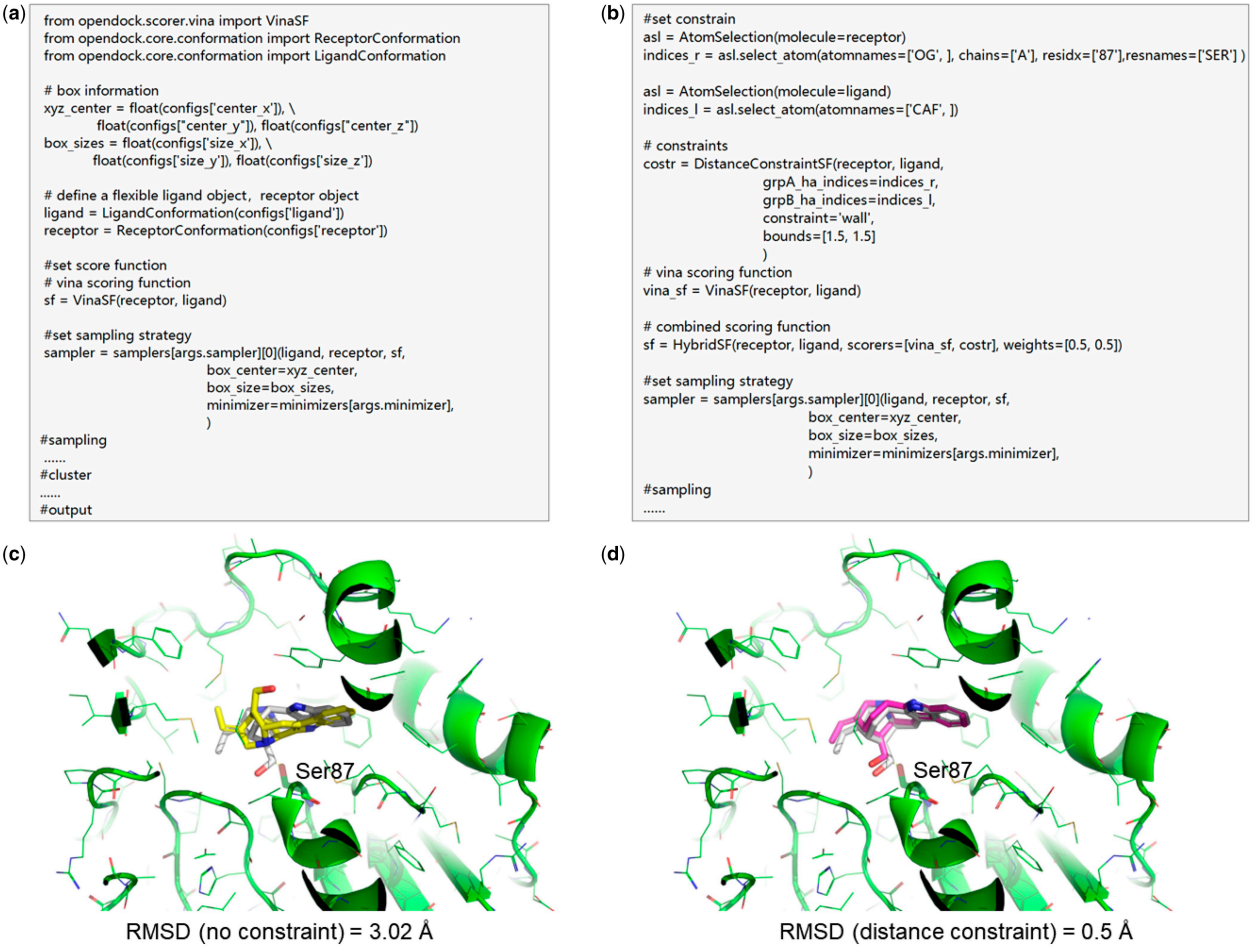
A docking example (PDBID:3GZJ) with/without distance constraint based on Vinascore scoring function. Panels (a and b) are the codes without constraints and with custom constraints, respectively. Panels (c) and (d) are the top-ranked pose of the ligand after docking without constraints and with constraints, respectively, where gray represents the native conformation, and yellow and pink represent the conformations after docking with OpenDock, and the distance is constrained between the Ser87 in the protein with carbonyl carbon atom in the ester bond of the molecule (EVS).

In this section, we implement a specific protein–ligand docking example both without constraints and using custom distance constraints to demonstrate the user-friendly nature of this framework (Fig. 7). Firstly, we define the pocket location and size, input a protein and a ligand ligand, and define the scoring function, which defaults to Vinascore. Then, we define the sampling strategy, proceed with sampling, and finally cluster and output the sampled conformations (Fig. 7a and b).

Using the case of polyneuridine aldehyde esterase (PDBID: 3GZJ (Yang *et al.* 2009)) as an example, we conducted a normal docking protocol without constraints where the scoring function is Vinascore. Due to the limited accuracy of the scoring function Vinascore (Trott and Olson 2010), the top-1 docking pose significantly differs from the native conformation (RMSD = 3.02 Å), as shown in Fig. 7c, where the docked conformation appears to be rotated 180° at the left side of the substrate molecule (EVS) compared to the native conformation. Relying solely on Vinascore is insufficient to resolve this issue.

To address this problem, we can use a custom distance constraint with the prior knowledge that the catalytic residue Ser87 of the esterase may attach to the carbonyl carbon of the ester bond in the substrate (EVS). As seen in Fig. 7b, a distance constraint of 1.5 Å is added between the OG atom of Ser87 side-chain of the esterase and the CAF atom (carbonyl carbon in the ester bond) of the ligand (EVS), forming a constrained scoring function: *Costr*. This *costr* is combined with Vinascore through *HybridSF* with equal weights of 0.5, thus forming a new scoring function.

As observed from Fig. 7d, by adding this distance constraint, the left-end discrepancy in the docked ligand conformation is effectively resolved, resulting in a top-1 pose very close to the native structure (RMSD = 0.5 Å). This process not only demonstrates the molecular docking workflow of OpenDock but also verifies the effectiveness of OpenDock’s custom constraints in solving specific problems, thereby providing robust technical support for future drug design and enzyme engineering. Notably, adding constraints increases computational overhead. Using 3GZJ as an example, docking without constraints took 50.0 s, adding distance constraints between atom pairs increased the time to 53.0 s, and applying the entire distance matrix constraint raised the time to 1568.8 s. The more constraints added, the higher the computational cost.

### 3.7 Future perspective

In OpenDock, various scoring functions and sampling strategies can be employed (Su *et al.* 2018, Shen *et al.* 2023). Based on the combinations of scoring functions and sampling strategies outlined in Tables 1 and 2, tests were conducted in different validation scenarios. By comparing the test results with the popular molecular docking framework AutoDock Vina, it is evident that our proposed OpenDock framework performs well in both validation scenarios (redocking and cross-docking).

It is worth noting that the results indicate that for a certain sampling strategy, using different scoring functions during post-processing can effectively improve the success rate (Ballester and Mitchell 2010, Zheng *et al.* 2022, Shen *et al.* 2023, Wang *et al.* 2023). Similarly, for the same scoring function, such as using Vinascore throughout the docking process, employing a more effective sampling strategy (such as PSO) can also significantly enhance the success rate. This underscores the importance of both scoring functions and sampling strategies in the molecular docking process.

Looking ahead, emerging deep learning methods like AlphaFold3 (Abramson *et al.* 2024), DiffDock (Corso *et al.* 2024), and the newly developed large language models(Huang and Li 2023), along with similar tools, hold great promise for advancing molecular docking. AlphaFold3 is expected to push the boundaries of protein structure prediction with higher precision and real-time modelling capabilities. Integrating AlphaFold3’s predictive models with OpenDock could potentially revolutionize the docking process by providing highly accurate protein–ligand complex predictions. The synergy between AlphaFold3’s structure prediction and OpenDock’s flexible sampling and scoring strategies could lead to faster and more reliable predictions of binding poses and affinities. Such an integration would enable us to further reduce the search space in docking, allowing for improved efficiency and accuracy in drug discovery pipelines.

OpenDock enables customization through the combination of various scoring functions and sampling strategies, which holds significant value for future exploration in molecular docking. With more precise protein–ligand distance matrix predictions (Dhakal *et al.* 2022; Lu *et al.* 2022; Stärk *et al.* 2022), the performance of protein–ligand modelling could be further improved by combining these predictions with OpenDock through the *Constraint* class.

The current framework, being based on PyTorch and Python, undeniably faces certain limitations in computational efficiency. Improving computational efficiency will be a key focus in future developments. However, this issue can be effectively mitigated by utilizing efficient sampling strategies and scoring functions. Additionally, the framework supports user customization of force field constraints through the *Constraint* class. This flexible molecular modelling approach allows for the effective reduction of the search space, thereby enhancing the efficiency and accuracy of molecular docking by setting appropriate intermolecular constraints.

## 4 Conclusion

In this work, we introduce OpenDock, a molecular docking framework based on PyTorch and Python. OpenDock allows for the utilization of various scoring functions and sampling strategies. Users can customize sampling strategies and integrate different scoring functions to achieve higher precision in molecular modelling. In both redocking and cross-docking tasks, OpenDock demonstrates superior performance in docking success rates. While it is not specifically designed to outperform AutoDock Vina, it provides users with the flexibility to develop complex docking protocols for various applications. Additionally, OpenDock supports user-defined constraints for covalent docking and local structure optimization. Overall, OpenDock is a highly useful tool for sophisticated protein–ligand modelling in drug discovery and enzyme design.

## Supplementary Material

btae628_Supplementary_Data
