# Supplementary material for: OpenDock: a pytorch-based open-source framework for protein–ligand docking and modelling
Source: Bioinformatics. 2024 Oct 21;40(11):btae628. doi: 10.1093/bioinformatics/btae628 (PMC11552628; doi:10.1093/bioinformatics/btae628)
Supplement: btae628_Supplementary_Data [file btae628_supplementary_data.pdf]

Supporting Materials

Part 1. Benchmark datasets

Table S1: Benchmark datasets used in performance comparison

Name	Description	Samples
CASF2016 coreset	Re-docking	285
PDBBind-Lowsim	Re-docking	249
Disco	Cross-Docking	4688
DockGen	Blind-Docking	189

Currently, there are two popular methods for evaluating molecular docking: redocking the original ligand back to its "native" receptor (Su et al. 2018) and docking a ligand into a non-native pocket (cross-docking) (Wierbowski et al. 2020). We validated OpenDock's redocking performance using the CASF2016 core set, which includes 285 protein-ligand complexes (Su et al. 2018). This dataset is commonly used as a benchmark for scoring functions, though it is also considered to be quite over-fitted (Su et al. 2020; Buttenschoen et al. 2024). Therefore, we introduced an additional similarity-clustered dataset, the PDBBind-Lowsim dataset, consisting of 249 complexes. This dataset was used as a standard test set in another study (Shen et al. 2023). The protein-ligand structures in this dataset were all released after January 1, 2021, and the protein sequences exhibit significant divergence from structures released prior to 2021 (with pairwise sequence identity less than 0.5). Consequently, these protein-ligand complexes were not "seen" by previous scoring functions, thereby mitigating the over-fitting problem to some extent (Su et al. 2020; Shen et al. 2023).

To evaluate the ability to dock a ligand into a non-native pocket, we used the Disco dataset (Wierbowski et al. 2020), excluding complexes that AutoDock Vina and OpenDock could not handle, such as those with specific atom types that cannot be processed. This resulted in 4,688 complexes for validating OpenDock's cross-docking performance.In addition, to further evaluate the ability to perform blind docking, we used the novel DockGen dataset, which consists of 189 complexes.

We compared the performance of OpenDock with the current most popular docking software, AutoDock Vina, by calculating the top-N success rate of finding a near-native pose (with RMSD less than 2 Å to the native ligand conformation) (Su et al. 2018) using the RMSD calculation tool obrms (O'Boyle et al. 2011).

Part 2. Comparison with AutoDock Vina and Parameter Exploration

Table S2: Parameters explored

Name	Description	Value explored
exhaustiveness	Number of chains in Monte Carlo sampling	4, 8, 16, 32
optimization step size	Number of steps for local optimization	5, 10, 15, 20

During the molecular docking process, OpenDock offers many adjustable parameters, such as exhaustiveness (number of chains in Monte Carlo sampling, defaulting to 8), boxsize (size of conformational exploration space, defaulting to 20), num_output (number of output conformations, defaulting to 20), min_rmsd_cluster (minimum RMSD to cluster saved poses, defaulting to 1.0), and parameters for local optimization like optimization step size (defaulting to 5) and learning rate (defaulting to 1.0). Most parameter settings align with the defaults of AutoDock Vina. Previous studies, like Gnina, have extensively explored the impacts of certain parameters, such as min_rmsd_cluster and num_output, and found their effects on results to be minimal. Therefore, the testing process of OpenDock will focus on exploring the impacts of exhaustiveness and local optimization step size on docking performance.

Comparing OpenDock with AutoDock Vina, both employ the Vinascore scoring function and utilize the Monte Carlo sampling strategy, aligning them in this aspect. Other parameters follow default settings. The exploration values of parameters are defined in Table S2, and each docking experiment generates a number of poses. These poses are processed using the Top N metric to indicate success rates, where a docking is considered successful if at least one pose among the top-N ranked poses has an RMSD value less than 2 Å.

First, we explored the size of the local optimization step. In AutoDock Vina (Trott and Olson 2010) and iDock (Li et al. 2012), L-BFGS (Moritz et al. 2016)-based minimization is adopted based on the grid-based energy surface, achieving very fast speeds. Unlike traditional docking frameworks, OpenDock employs an Adam-based minimizer (an inherited class of the sampler) for optimizing ligand conformations, allowing for the use of various differentiable scoring functions, including both traditional (Trott and Olson 2010; Masters et al. 2020) and deep learning-based methods (Wang et al. 2023). Two crucial parameters in this process

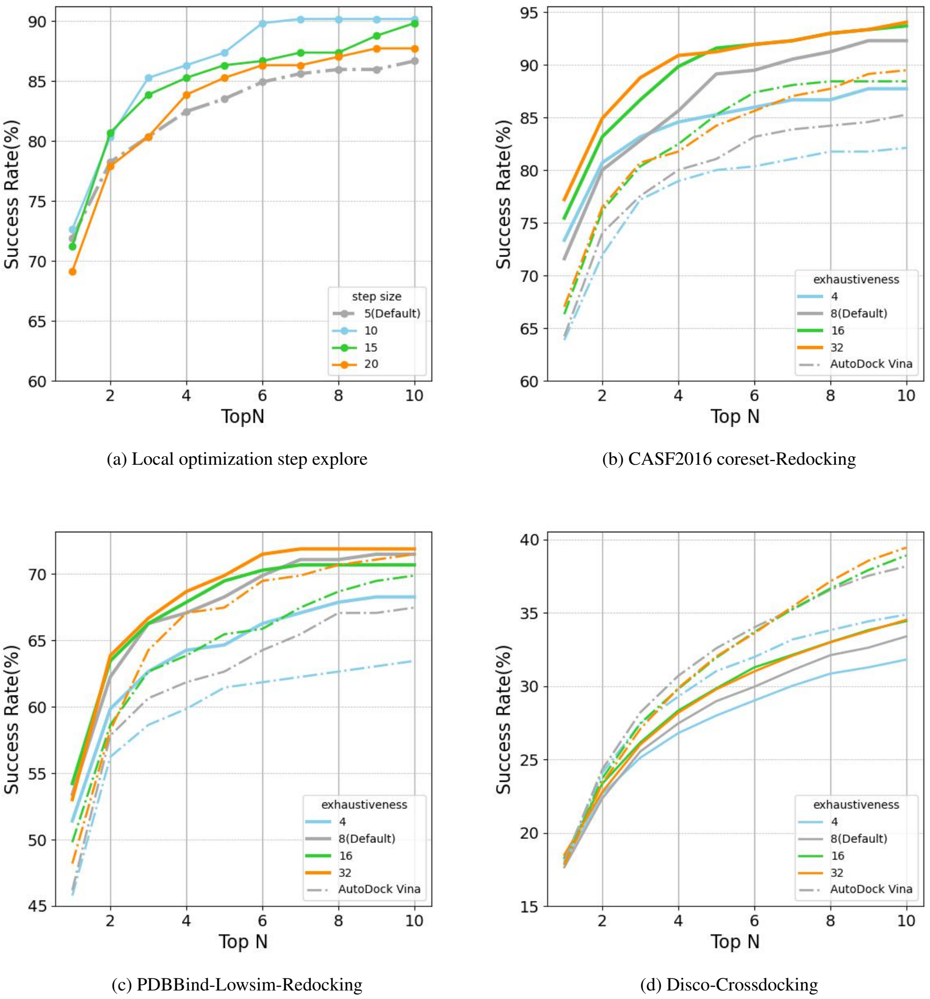

(a) Local optimization step explore

(b) CASF2016 coreset-Redocking

(c) PDBBind-Lowsim-Redocking

(d) Disco-Crossdocking

Figure S1: Parameter exploration. (a) Using Monte Carlo sampling with an exhaustiveness setting of 4 (4MC). The dataset is CASF2016 coreset, and the validation scenario is redocking testing. This is the result obtained by using different local optimization step sizes. (b-d) Validating redocking and crossdocking across three datasets, utilizing Monte Carlo sampling with different exhaustiveness settings. Dashed lines represent the AutoDock Vina test results, while solid lines represent the OpenDock test results.Top N is the percentage of targets ranked above or at N with a RMSD value less than 2 Å.

are the learning rate and optimization step size. After adjustments, we found that a learning rate of 0.1 yields better results, thus setting it as the default. The optimization step size is a parameter worth further exploration. With exhaustiveness set to 4, we tested local optimization step sizes of 5, 10, 15, and 20 on the CASF2016 core set for redocking, and the results are shown in Figure S1a. Interestingly, as the local optimization step size increases, the docking success rate does not exhibit a linear growth. This indicates that the minimizer can quickly complete the local pose optimization. Considering both time cost and performance, setting the local optimization step size to 5 is the most efficient. Hence, we set the local optimization step size to 5 for the following experiments.

Next, we explored the impact of different exhaustiveness values on the redocking and cross-docking tasks. Considering the consumption of computational resources and time, we set the maximum exhaustiveness value to 32. In the validation tests conducted on three datasets for redocking and cross-docking, the results (shown in Figures S1b, S1c, S1d) indicate that as exhaustiveness increases, the improvement in performance does not exhibit linear growth with the increase in sampling efforts. Both OpenDock and AutoDock Vina exhibit the same trend. Specifically, in redocking tasks, OpenDock demonstrated a significant improvement in success rate compared to AutoDock Vina, and it also performed well in cross-docking. Considering time costs, we set the default exhaustiveness value to 8. It is reasonable to use a maximum exhaustiveness value of 32 in subsequent experiments.

Part 3. Description and comparison of the features of current popular docking tools

There are currently many traditional molecular docking software programs available. Here we list the sampling and scoring strategies used by these methods or applications in Table S3.

Table S3: Summary of Traditional Docking Tools

Docking Tool	Scoring Function	Sampling Strategy
AutoDock Vina(Trott and Olson 2010)	Combines knowledge-based and force-field terms; evaluates both inter-molecular and intra-molecule energies as well as ligand torsional angle normalization term.	Uses global exploration via Monte Carlo algorithms and local optimization using L-BFGS along pre-calculated grids.
Smina(Koes et al. 2013)	Extends Vina's scoring with different energy weighting parameters.	Similar to Vina with enhanced Monte Carlo for global searches and advanced local optimization tailored to docking specifics.
Glide(Friesner et al. 2004)	Hybrid scoring combining molecular mechanics energy and empirical terms.	Uses a hierarchical approach starting with fast conformational sampling then refined searching.
FlexX(Rarey et al. 1996)	Knowledge-based scoring using atom-atom potential energy terms focusing on Van der Waals, hydrogen bonding, and hydrophobic interactions.	Uses an incremental construction algorithm, building the protein-ligand complex by adding interaction points while considering ligand flexibility.
Gold(Jones et al. 1997)	Cheminformatics-based and empirical force-field terms; evaluates protein-ligand complementarity, hydrogen bonding (used for genetic algorithm searching), hydrophobic interactions, and ligand strain.	Genetic algorithm-based strategy exploring conformational space systematically. Multiple genetic islands would be adopted for docking pose diversities.

Except for the traditional methods, more recent deep learning based molecular docking methods mostly focus on global docking with very large ligand binding space searching with graph-based scoring modules but not as explicit as the scoring functions adopted in traditional methods, see Table S4.

Part 4. Introducing and analyzing various sampling strategies in OpenDock

In addition to Monte Carlo (MC) sampling, OpenDock offers multiple custom sampling strategies. In the Genetic Algorithm (GA) sampling strategy (Mirjalili and Mirjalili 2019), each conformation represented by the (6+k) vector is encoded into a chromosome, which can be easily decoded back for energy evaluation. A population size is set, and multiple individuals can undergo crossover and mutation. Following crossover and mutation, local optimizations by the minimizer are conducted to optimize the docking poses in the population, resulting in the continuous evolution of new populations. The Particle Swarm Optimization (PSO) strategy (Wang et al. 2018) operates on a similar principle, continuously mutating and locally optimizing while allowing each individual to learn from the current optimal individual in a niche and the global optimum, updating the population accordingly.

Using a specific example (PDBID: 1gpn), we investigate how the ligand scores and RMSD evolve with increasing sampling steps for three different sampling strategies. To better illustrate this change, the scores are increased by 20 (to ensure they are greater than 0) and then logarithmically transformed. The population size is set to 3 for PSO and 4 for GA. Given that the selected case is not very complex, the step limit is set to 50 and divided into two parts for more detailed analysis. From figure S2, it can be seen that due to the significant deviation between the initial ligand conformation and the natural conformation, there is a notable decrease in scores from step 0 to 10. In general, the RMSD tends to decrease as well in strategies like MC and PSO(S2a,S2c), but this is not absolute, as in GA(S2e), where the RMSD shows a trend of increase as the scores decrease. From step 10 to 50, the distinct characteristics of different sampling strategies become evident. In MC sampling(S2b), the scores and RMSD exhibit erratic back-and-forth movements, which align with the nature of MC sampling. In contrast, PSO and GA(S2d,S2f), due to the mutual learning among multiple individuals, show a clear downward trend in both scores and RMSD.

In terms of sampling time, the sampling durations for GA and PSO in redocking and cross-docking tests are comparable to that of 4MC (MC sampling with exhaustiveness set to 4). For a detailed analysis of time efficiency, refer to Figure S6.

Table S4: Summary of Advanced Docking Tools

Docking Tool	Scoring Function	Sampling Strategy
Gnina(McNutt et al. 2021)	Utilizes a deep learning-based scoring function (3D-CNN) integrating protein structure, ligand structure, and protein-ligand interaction patterns, predicting binding affinity and binding probability using a multi-task learning framework.	Combines a Monte Carlo sampling algorithm for global searching with gradient-based scoring function optimization for local searching as does in AutoDock Vina.
EquiBind(Stärk et al. 2022)	Employs a SE(3)-equivariant geometric model to represent molecular structures and interactions, utilizing residue-level and atom-level representations to predict binding site and ligand pose (position and orientation).	Ligand poses are generated with distance geometric constraints with fast ligand fitting with torsional angle manipulation to allow for ligand flexibility and to save computation time.
TankBind(Lu et al. 2022)	Trigonometry-aware neural networks are constructed to predict the binding affinity (assuming that the native pose affinity is higher than the docking poses) and protein-ligand distance map.	Iterative updated ligand coordinates are optimized by the distance map between the protein and the ligand, and this distance map considers both the inter-molecule orientations and also the ligand conformation reliability (or validity).
DiffDock(Corso et al. 2024)	Utilizes a deep learning approach based on a diffusion model to minimize an upper bound on the negative log-likelihood of the observed structures under the model's distribution. A confident score (which indicates the possibility of a pose with the difference tolerance to the native pose) model is trained to rank the generated poses.	Driven by the diffusion model's generative process, it generates possible ligand conformations (including translations of ligand position, rigid global rotation and ligand internal torsional rations) that are progressively refined through denoising steps.

Part 5. Analysis of the computational cost involved in the sampling routines

In order to analyze the sampling strategies and their corresponding computational costs in detail, we will examine the pseudocode of each sampling strategy . The computational overhead during the sampling process is mainly attributed to local optimizations, as it is implemented based on neural networks. Therefore, the efficiency of the three sampling strategies is also related to the total number of local optimization steps. As shown in the pseudocode (algorithm 1,2,3), the sampling depth of Genetic Algorithm (GA) and Particle Swarm Optimization (PSO) can be assessed by their level of exhaustiveness, similar to Monte Carlo (MC) methods. While GA and PSO introduce additional computational overhead due to the interactions among various individuals, the majority of these costs are primarily associated with the number of local optimizations.

In comparison with AutoDock Vina, since OpenDock's code is written in Python and uses neural network-based local optimization, directly comparing the total runtime of OpenDock and AutoDock Vina does not accurately reflect their sampling and search efficiencies. To further evaluate their sampling efficiencies, we analyze the relationship between the total number of sampling steps and the success rate. For a given ligand and a set exhaustiveness, the total number of sampling steps is fixed for both OpenDock and AutoDock Vina. Since OpenDock operates at a slower speed, the total number of steps it defines is fewer than those of AutoDock Vina. Specifically, the total number of steps in OpenDock and AutoDock Vina(as seen from the source code) are:

$$\text{O\_step} = 100 \times \text{num\_atom} \times \text{exhaustiveness}. \tag{1}$$

$$\text{heuristic} = \text{movable\_atoms} + 10 \times \text{degrees\_of\_freedom}. \tag{2}$$

$$\text{V\_steps} = \frac{70 \times 3 \times (50 + \text{heuristic})}{2} \times \text{exhaustiveness} . \tag{3}$$

Where O_step represents the total steps of OpenDock, V_steps represents the total steps of AutoDock Vina, num_atom rep-

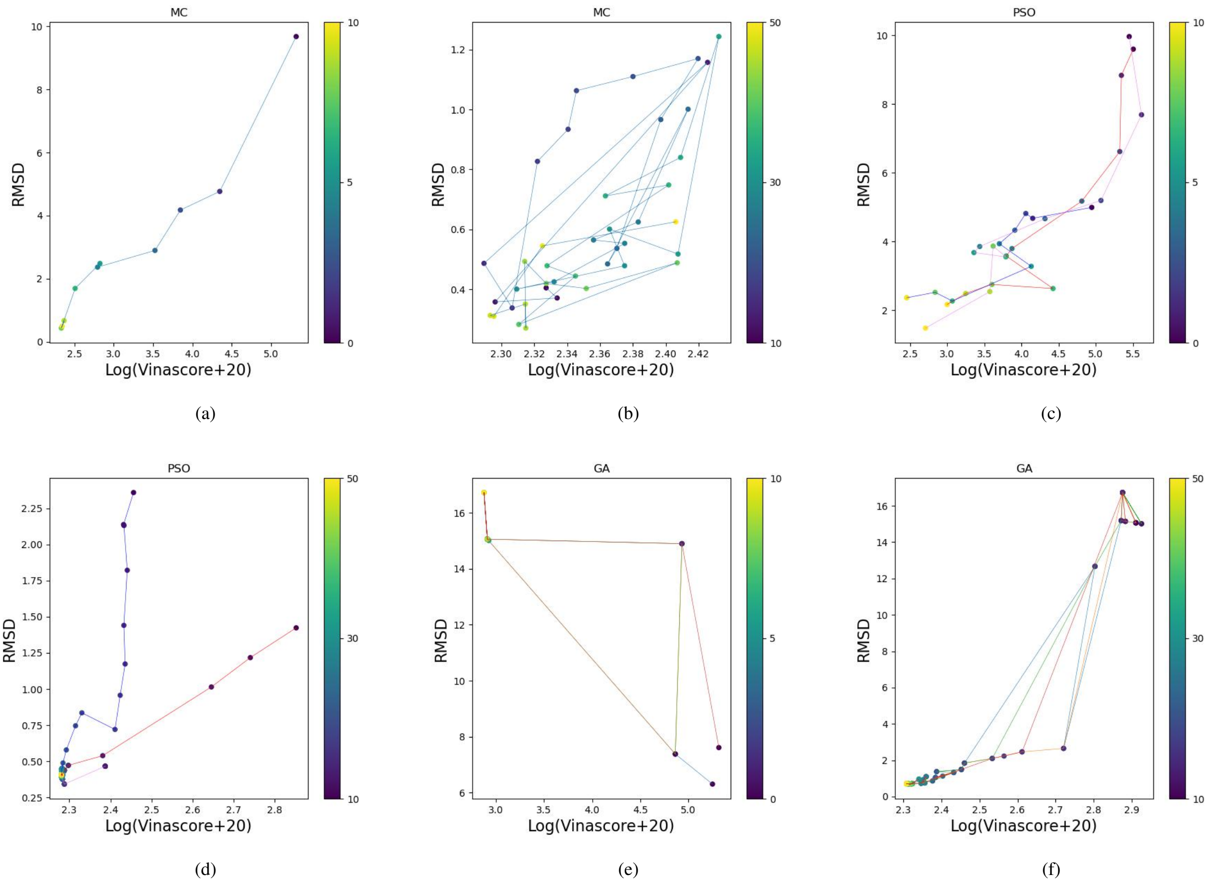

Figure S2: Ligand scores and RMSD evolve with increasing sampling steps for three different sampling strategies.Total steps are set to 50, with a PSO population size of 3 and a GA population size of 4. **a, b**: MC sampling process, **c, d**: PSO sampling process, **e, f**: GA sampling process.In PSO and GA, lines of different colors connect different individuals in the population.

resents the number of heavy atoms in the ligand, movable_atoms represents the number of movable atoms in the ligand, and degrees_of_freedom refers to the ligand's degrees of freedom. From the above formulas, we can derive the following:

$$\text{V\_steps} > 100 \times 60 \times \text{exhaustiveness} \, . \tag{4}$$

In most cases, when the ligand has fewer than 60 heavy atoms, the total sampling steps in OpenDock are fewer than those in AutoDock Vina. Therefore, we conclude that OpenDock performs better than AutoDock Vina in the redocking tests.

To compare the performance of different sampling strategies in OpenDock, we kept the total number of sampling steps consistent for MC, GA, and PSO by setting the same exhaustiveness for all. In the redocking and cross-docking tests, the exhaustiveness for PSO and GA was set to a minimum value of 4. Despite this, improvements were still observed compared to MC with higher exhaustiveness in the redocking tests. Therefore, we can conclude that the sampling strategies of GA and PSO offer improvements over MC in the redocking tests. However, in the DockGen tests, where the initial structures deviate significantly from the crystal structure, GA and PSO exhibited disadvantages.This also demonstrates that different sampling strategies have their own advantages in different scenarios.

Part 6. Parallelism with multiple CPU cores

To fully utilize CPU resources and implement multiprocessing parallel sampling, we adopted the pattern of iDock (Li et al. 2012) and introduced a multiprocessing mode, considering the associated time overhead. For Monte Carlo sampling, we reduced the number of steps for each Monte Carlo chain while increasing the number of Monte Carlo samples, thereby maintaining the total sampling overhead consistent with the sampling patterns in iDock. By default, the number of steps for each individual sample is set to 100 times the number of heavy atoms in the ligand.

Algorithm 1 Monte Carlo Sampling for Protein-Ligand Docking

Require: *receptor*, ligand, $MC(sampler)$,scorer,$exhaustiveness$
Ensure: Implemented local optimization

$total\_steps \Leftarrow exhaustiveness \times num\_heavy\_atoms \times 100$
Generate multiple sampling chains based on total_steps
for each chain **do**
    $current\_conf \Leftarrow$ random initial conformation
    for i = 1 to $steps$ **do**
        $new\_conf \Leftarrow$ random perturbation of $current\_conf$
        $new\_conf \Leftarrow$ local optimization of new_conf
        if $Score(new\_conf) > Score(current\_conf)$ **then**
            $current\_conf \Leftarrow new\_conf$
        else
            Accept new_conf with probability $P(\Delta score)$
        end if
    end for
end for
Aggregate all chains, cluster and re-rank conformations
return Top-ranked conformations

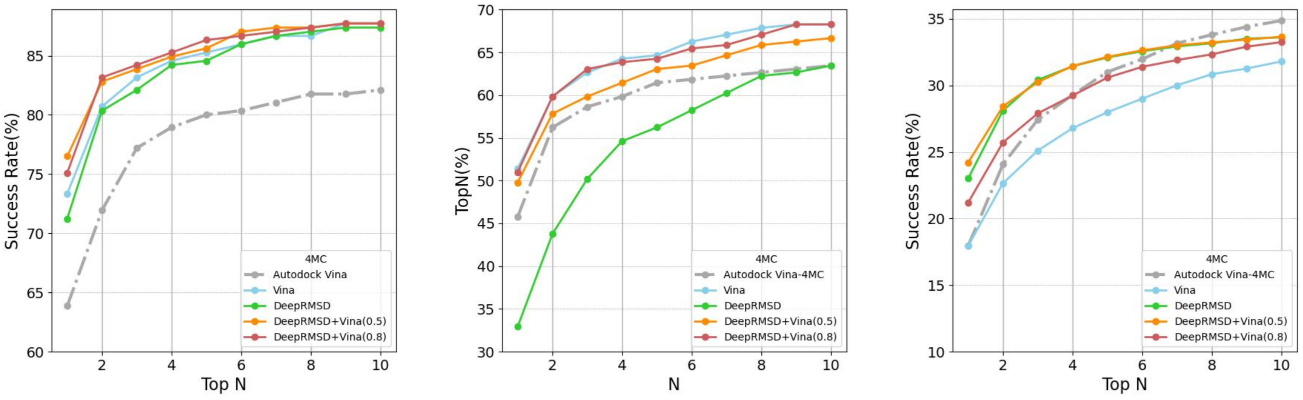

(a) CASF2016 coreset-Redocking (b) PDBBind-Lowsim-Redocking (c) Disco-Crossdocking

Figure S3: Using Monte Carlo sampling with an exhaustiveness setting of 4 (4MC), different scoring functions were employed for post-processing. DeepRMSD+Vina(0.5) indicates that the weight of Vina score is set to 0.5.Top N is the percentage of targets ranked above or at N with a RMSD less than 2 Å.

Part 7. Blind Docking with Deviated Ligand Conformations

In addition to the crystal structure conformation, we also evaluated docking performance using ligand conformations that deviate significantly from the crystal structure, generated by AutoDock Vina. This approach aimed to assess the robustness of the docking tools under conditions where the ligand input is less accurate.

However, from the results in Table S5, when using conformations that deviate significantly from the crystal structure as input, the performance of tools like AutoDock Vina and Smina remained unchanged. In contrast, DiffDock-L, Gnina, and OpenDock exhibited a certain degree of performance decline. For OpenDock, increasing the sampling depth somewhat alleviated the performance drop. Among the sampling strategies, GA and PSO exhibited the most significant decrease compared to MC. This may be attributed to the large deviation between the initial conformation and the crystal structure, where the neural network-based local optimization tends to get trapped in local minima. The learning interactions between population members in GA and PSO might have exacerbated this issue, leading to a further drop in success rates. Therefore, in such scenarios, the random sampling strategy employed by MC proved more advantageous, highlighting the different characteristics of sampling strategies in various contexts. This finding underscores the potential for further exploration in optimizing sampling strategies based on specific scenarios.

Algorithm 2 Genetic Algorithm Sampling for Protein-Ligand Docking

Require: $receptor$,ligand,$GA(sampler)$,scorer,$exhaustiveness$, $population\_size$,mutation_rate,$crossover\_rate$
Ensure: Implemented local optimization

```
total_steps ⇐ exhaustiveness × num_heavy_atoms × 100
Generate multiple populations based on total_steps and population_size
for each population do
    for step = 1 to steps do
        for each individual in population do
            new_conf ⇐ mutation (random perturbation) with probability mutation_rate
            new_conf ⇐ local optimization of new_conf
            if Score(new_conf) > Score(current_conf) then
                current_conf ⇐ new_conf
            else
                Accept new_conf with probability P(Δscore)
            end if
        end for
        for each pair of individuals selected for crossover do
            new_conf ⇐ crossover to exchange information between individuals with probability crossover_rate
            new_conf ⇐ local optimization of new_conf
            if Score(new_conf) > Score(current_conf) then
                current_conf ⇐ new_conf
            else
                Accept new_conf with probability P(Δscore)
            end if
        end for
    end for
end for
Aggregate all populations, cluster and re-rank conformations
return Top-ranked conformations
```

Part 8. Results of other exhaustiveness values for Monte Carlo sampling across various scoring functions

Results for different Monte Carlo sampling exhaustiveness values (4MC:Figure S3, 16MC:Figure S4, 32MC:Figure S5) using various scoring functions, such as Vina and Vina+DeepRMSD, in both redocking and cross-docking validation scenarios.

Part 9. Computational efficiency

The computational efficiency of docking protocols is crucial for large-scale virtual screening. Due to its implementation based on Python (Van Rossum and Drake Jr 1995) and PyTorch (Paszke et al. 2019), OpenDock may lag in terms of time efficiency compared to molecular docking frameworks like AutoDock Vina. However, in the current multi-core CPU parallel processing paradigm, decomposing individual docking tasks into multiple parallel tasks can significantly reduce the overall task completion time.

For example, in the redocking task of the CASF2016 core set dataset, using the Monte Carlo sampling strategy with an exhaustiveness setting of 4 (4MC), docking calculations were performed using 16 CPU cores on a Linux cluster system equipped with Intel(R) Xeon(R) Gold 5220R CPUs @ 2.20GHz. The calculation time consumption is illustrated in the supporting materials(Figure S6). On average, OpenDock consumes approximately 2.46 minutes per docking task. As the number of rotatable bonds in the ligand increases, the docking time also increases. The docking time consumption for sampling strategies using GA, PSO, and Monte Carlo (4MC) are similar, with average times of 2.93 minutes, 2.25 minutes, and 2.46 minutes, respectively.

Algorithm 3 Particle Swarm Optimization Sampling for Protein-Ligand Docking

Require: *receptor*,ligand,*PSO*(*sampler*),scorer,*exhaustiveness*, *population_size*,*social_param*
Ensure: Implemented local optimization

```
total_steps ⇐ exhaustiveness × num_heavy_atoms × 100
Generate multiple populations based on total_steps and population_size
for each population do
    initialize best_conf
    for step = 1 to steps do
        for each individual in population do
            new_conf ⇐ random perturbation of current_conf
            new_conf ⇐ local optimization of new_conf
            if Score(new_conf) > Score(current_conf) then
                current_conf ⇐ new_conf
            else
                Accept new_conf with probability P(Δscore)
            end if
            if Score(current_conf) > Score(best_conf) then
                best_conf⇐ current_conf
            end if
            new_conf ⇐ current_conf learns from best_conf according to social_param
            new_conf ⇐ local optimization of new_conf
            if Score(new_conf) > Score(current_conf) then
                current_conf ⇐ new_conf
            else
                Accept new_conf with probability P(Δscore)
            end if
        end for
    end for
end for
Aggregate all populations, cluster and re-rank conformations
return Top-ranked conformations
```

Table S5: Top-1 RMSD performance of different methods on the DOCKGEN benchmarks with deviated conformations input. The values in parentheses refer to the level of exhaustiveness of the search; for example, (8) indicates that the exhaustiveness was set to 8.

Method	% <2Å	% <5Å	CPU(s)	GPU(s)
P2rank+AutoDock Vina(8)[1]	17.5	37.0	15.1	-
P2rank+AutoDock Vina(32)[1]	**17.5**	37.6	26.3	-
P2rank+Smina(8)[1]	15.9	33.9	**13.7**	-
P2rank+Smina(32)[1]	16.4	36.0	29.7	-
P2rank+Gnina(8)[1]	13.8	27.0	137.3	-
P2rank+Gnina(32)[1]	17.5	35.0	147.0	-
P2rank+OpenDock-MC(8)[1]	12.7	28.0	197.1	-
P2rank+OpenDock-MC(32)[1]	14.8	30.2	884.2	-
P2rank+OpenDock-GA(8)[1]	11.6	26.5	368.4	-
P2rank+OpenDock-GA(32)[1]	13.8	27.5	1384.2	-
P2rank+OpenDock-PSO(8)[1]	11.1	27.0	315.8	-
P2rank+OpenDock-PSO(32)[1]	15.3	31.2	1214.7	-
Equibind	0.0	4.8	-	**0.04**
Tankbind	0.5	16.9	-	0.7
DiffDock-L	16.4	**52.9**	246.3	25

Smina and Gnina run according to default settings.
[1] Assign 24 CPU cores to each task.

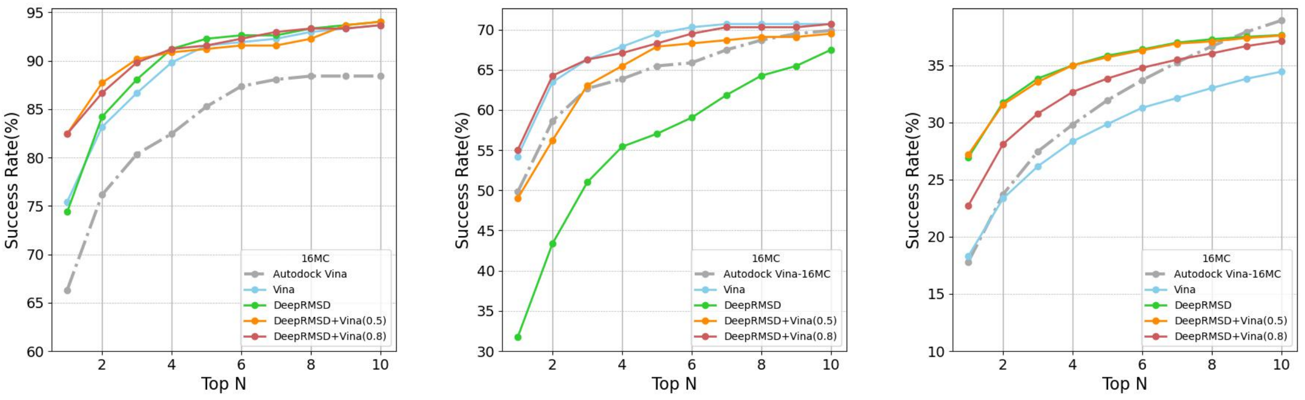

(a) CASF2016 coreset-Redocking (b) PDBBind-Lowsim-Redocking (c) Disco-Crossdocking

Figure S4: Using Monte Carlo sampling with an exhaustiveness setting of 16 (16MC), different scoring functions were employed for post-processing. DeepRMSD+Vina(0.5) indicates that the weight of Vina score is set to 0.5.Top N is the percentage of targets ranked above or at N with a RMSD less than 2 Å.

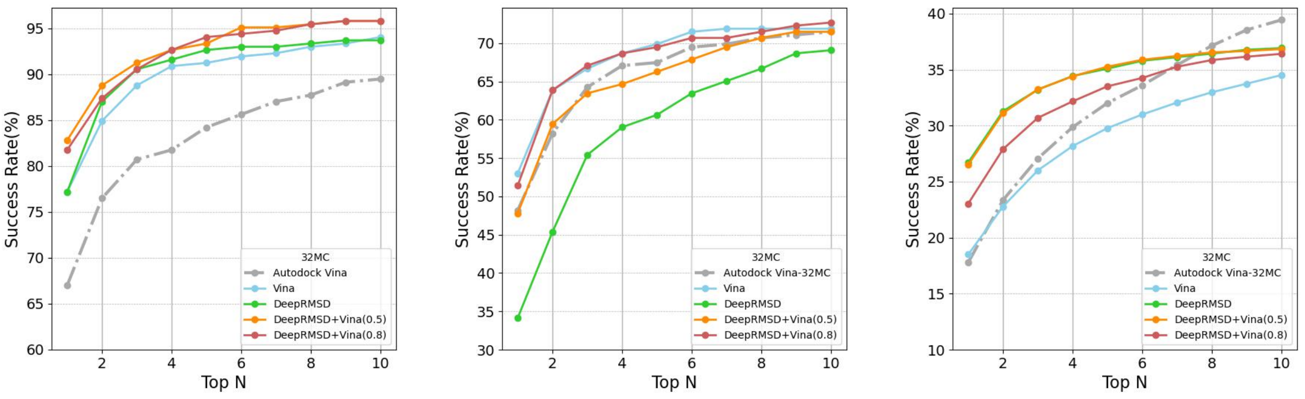

(a) CASF2016 coreset-Redocking (b) PDBBind-Lowsim-Redocking (c) Disco-Crossdocking

Figure S5: Using Monte Carlo sampling with an exhaustiveness setting of 32 (32MC), different scoring functions were employed for post-processing. DeepRMSD+Vina(0.5) indicates that the weight of Vina score is set to 0.5.Top N is the percentage of targets ranked above or at N with a RMSD less than 2 Å.

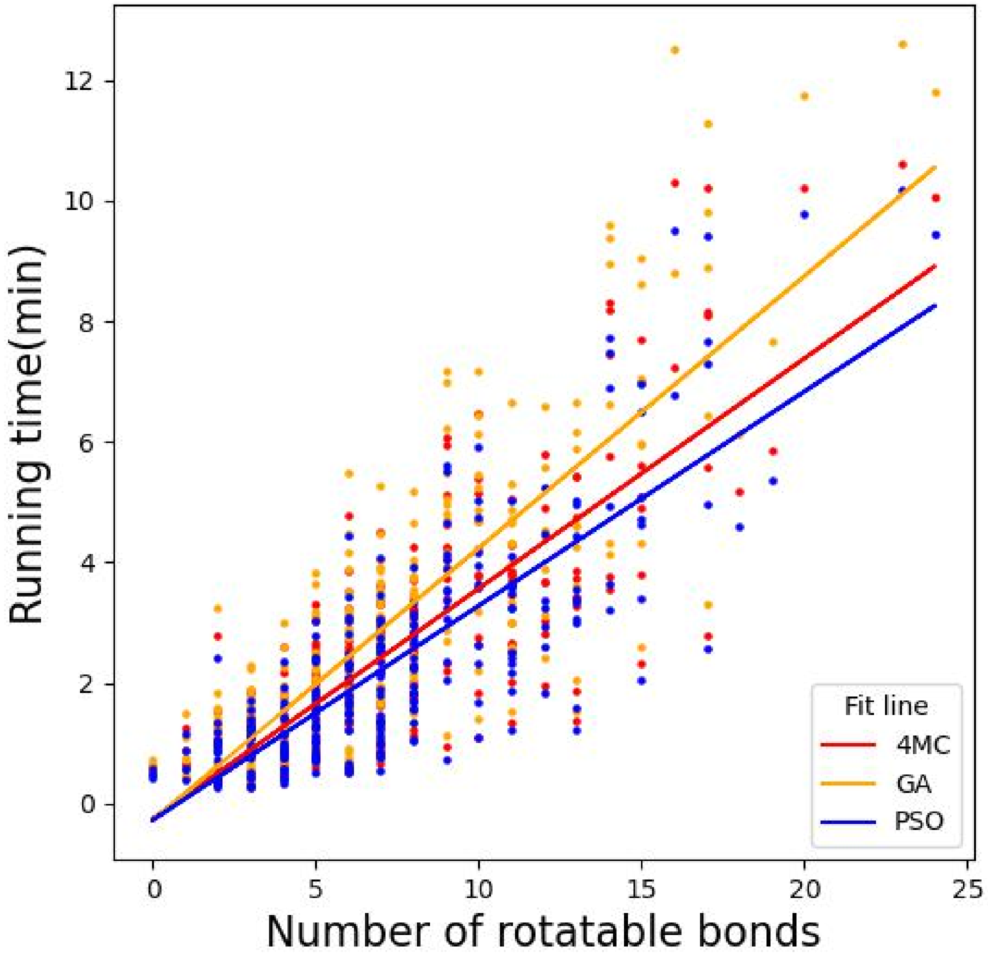

Figure S6: The relationship between the running time of Opendock and the number of rotatable bonds of ligands in the CASF2016 coreset. The red line, orange line, and blue line represent the linear fitting of MC, GA, and PSO sampling strategies, respectively.4MC stands for Monte Carlo sampling with an exhaustiveness setting of 4.

References

Buttenschoen, M., Morris, G. M., and Deane, C. M. (2024). Posebusters: Ai-based docking methods fail to generate physically valid poses or generalise to novel sequences. Chemical Science.

Corso, G., Deng, A., Polizzi, N., Barzilay, R., and Jaakkola, T. (2024). Deep confident steps to new pockets: Strategies for docking generalization. In International Conference on Learning Representations (ICLR).

Friesner, R. A., Banks, J. L., Murphy, R. B., Halgren, T. A., Klicic, J. J., Mainz, D. T., Repasky, M. P., Knoll, E. H., Shelley, M., Perry, J. K., et al. (2004). Glide: a new approach for rapid, accurate docking and scoring. 1. method and assessment of docking accuracy. Journal of medicinal chemistry, 47(7):1739–1749.

Jones, G., Willett, P., Glen, R. C., Leach, A. R., and Taylor, R. (1997). Development and validation of a genetic algorithm for flexible docking. Journal of Molecular Biology, 267(3):727–748.

Koes, D. R., Baumgartner, M. P., and Camacho, C. J. (2013). Lessons learned in empirical scoring with smina from the csar 2011 benchmarking exercise. Journal of Chemical Information and Modeling, 53(8):1893–1904.

Li, H., Leung, K.-S., and Wong, M.-H. (2012). idock: A multithreaded virtual screening tool for flexible ligand docking. In 2012 IEEE Symposium on Computational Intelligence in Bioinformatics and Computational Biology (CIBCB), pages 77–84. IEEE.

Lu, W., Wu, Q., Zhang, J., Rao, J., Li, C., and Zheng, S. (2022). Tankbind: Trigonometry-aware neural networks for drug-protein binding structure prediction. bioRxiv, pages 2022–06.

Masters, L., Eagon, S., and Heying, M. (2020). Evaluation of consensus scoring methods for autodock vina, smina and idock. Journal of Molecular Graphics and Modelling, 96:107532.

McNutt, A. T., Francoeur, P., Aggarwal, R., Masuda, T., Meli, R., Ragoza, M., Sunseri, J., and Koes, D. R. (2021). Gnina 1.0: molecular docking with deep learning. Journal of cheminformatics, 13(1):1–20.

Mirjalili, S. and Mirjalili, S. (2019). Genetic algorithm. Evolutionary algorithms and neural networks: Theory and applications, pages 43–55.

Moritz, P., Nishihara, R., and Jordan, M. (2016). A linearly-convergent stochastic l-bfgs algorithm. In Artificial Intelligence and Statistics, pages 249–258. PMLR.

O'Boyle, N. M., Banck, M., James, C. A., Morley, C., Vandermeersch, T., and Hutchison, G. R. (2011). Open babel: An open chemical toolbox. Journal of cheminformatics, 3:1–14.

Paszke, A., Gross, S., Massa, F., Lerer, A., Bradbury, J., Chanan, G., Killeen, T., Lin, Z., Gimelshein, N., Antiga, L., et al. (2019). Pytorch: An imperative style, high-performance deep learning library. Advances in neural information processing systems, 32.

Rarey, M., Böhm, H.-J., Steffen, T., and Thomas, L. (1996). A fast flexible docking method using an incremental construction algorithm. Journal of Molecular Biology, 261(3):470–489.

Shen, T., Liu, F., Wang, Z., Sun, J., Bu, Y., Meng, J., Chen, W., Yao, K., Mu, Y., Li, W., et al. (2023). zposescore model for accurate and robust protein–ligand docking pose scoring in casp15. Proteins: Structure, Function, and Bioinformatics, 91(12):1837–1849.

Stärk, H., Ganea, O., Pattanaik, L., Barzilay, R., and Jaakkola, T. (2022). Equibind: Geometric deep learning for drug binding structure prediction. In International Conference on Machine Learning, pages 20503–20521. PMLR.

Su, M., Feng, G., Liu, Z., Li, Y., and Wang, R. (2020). Tapping on the black box: how is the scoring power of a machine-learning scoring function dependent on the training set? Journal of chemical information and modeling, 60(3):1122–1136.

Su, M., Yang, Q., Du, Y., Feng, G., Liu, Z., Li, Y., and Wang, R. (2018). Comparative assessment of scoring functions: the casf-2016 update. Journal of chemical information and modeling, 59(2):895–913.

Trott, O. and Olson, A. J. (2010). Autodock vina: improving the speed and accuracy of docking with a new scoring function, efficient optimization, and multi-threading. Journal of computational chemistry, 31(2):455–461.

Van Rossum, G. and Drake Jr, F. L. (1995). Python tutorial.

Wang, D., Tan, D., and Liu, L. (2018). Particle swarm optimization algorithm: an overview. Soft computing, 22:387–408.

Wang, Z., Zheng, L., Wang, S., Lin, M., Wang, Z., Kong, A. W.-K., Mu, Y., Wei, Y., and Li, W. (2023). A fully differentiable ligand pose optimization framework guided by deep learning and a traditional scoring function. Briefings in Bioinformatics, 24(1):bbac520.

Wierbowski, S. D., Wingert, B. M., Zheng, J., and Camacho, C. J. (2020). Cross-docking benchmark for automated pose and ranking prediction of ligand binding. Protein Science, 29(1):298–305.
